# Postoperative complications following tonsil and adenoid removal in Kuwaiti children: A retrospective study

**DOI:** 10.1016/j.amsu.2018.09.024

**Published:** 2018-09-24

**Authors:** Khalid Al Sebeih, Jumana Hussain, Ahmed N. Albatineh

**Affiliations:** a ENT Surgeon, ENT Department, AL- Farwaniya Institute Kuwait, Kuwait; b Consultant of Otolaryngology Head and Neck Surgery, Fascial Plastics, Kuwait University, Kuwait; c Department of Community Medicine and Behavioral Sciences, Faculty of Medicine, Kuwait University, Kuwait

**Keywords:** Adenoidectomy, Adenotonsillectomy, Tonsillectomy, OR, Odds ratio, PV, Probability value, URTI, Upper respiratory tract infection

## Abstract

**Background:**

Adenoidectomy and adenotonsillectomy are two of the most common procedures that are performed by otolaryngologists around the world. Complications, ranging from major to minor ones, are affected by the preoperative symptoms and health status of the patient. We aimed to identify the prevalence of major postadenoidectomy and adenotonsillectomy complications, including bleeding, and minor complications, including malodor, fever, and snoring.

**Materials and methods:**

We conducted a retrospective chart review of 825 patients who underwent surgery between January 2002 and 30 December 2016 at our institution.

**Results:**

The bleeding complications prevalence was 4.1% (14/344) among patients with adenotonsillectomy and 1.3% (6/480) among those who underwent adenoidectomy. Results revealed that tonsil grade 3 patients were at a reduced risk (86% reduced risk) of developing bleeding complications, compared to those with tonsil grade 2 [odds ratio (OR) = 0.141, 95% confidence interval (CI): (0.028, 0.715)]. Grade C tympanogram patients had ten times the odds of bleeding complications compared to those with tympanogram grade A [OR = 10.6, 95% CI: 0.917, 122.54], a marginally significant difference (probability value (*PV*) = 0.054). Upper respiratory tract infections (URTIs) patients had three times the odds of bleeding complications compared to those without URTIs [OR = 3.03, 95% CI: (0.979, 9.439)], also a marginally significant difference (PV = 0.055). Postoperatively, 71% experienced no malodor, 23% had malodor lasting 3–7 days, and 1% had malodor for 7–10 days. Our analysis showed that 71% of the patients did not complain of snoring, 25% had snoring for 3–7 days, and 2% had snoring for 7–10 days. 80% of the patients did not develop fever, 13% had fever for 3–7 days, and no patients experienced fever for longer than 7 days.

**Conclusions:**

Nearly 4% of the patients developed bleeding after adenotonsillectomy and only 2% of the patients had only bleeding after adenoidectomy. Conversely, 15–25% of the patients developed minor complications, including malodor, snoring, and fever, independent of their preoperative symptoms.

## Highlights

• Adenoidectomy and adenotonsillectomy are two of the most common procedures that are carried out in the world.
• Primary and secondary hemorrhages are major complications.
• Aim of our study is to determine the prevalence of postadenoidectomy or adenotonsillectomy bleeding and minor complications.
• None of the previous studies reviewed minor complications that patients experience during the first few days postoperatively.

## 1 Introduction

Adenoidectomy and adenotonsillectomy are two of the most common procedures that are carried out by otolaryngologists especially among pediatricians, with an annual rate of approximately 250,000 cases [1].

Adenoidectomy is often correlated with additional surgical procedures, including tonsillectomy, or placement of tympanostomy tubes, and most tonsillectomies are usually performed in conjunction with adenoidectomies. Despite the vast body of adenoid-focused research, debate remains concerning the indications for adenoidectomy.

Primary and secondary hemorrhages are major complications for all patients undergoing adenoidectomy and adenotonsillectomy. The first complication is immediate bleeding, which arises during the procedure. This is considered rare as it occurs in only 0.4% of the cases. Major bleeding requiring operating room transfer occurs in 4 out of 1000 patients [2]. Significant delayed bleeding is observed in roughly 2% of patients undergoing tonsillectomy, but it is not generally observed with adenoidectomy [2].

Dehydration and refractory emesis are complications that can be alarming in pediatric patients because of their reduced hemodynamic reserves [3]. Major complications include velopharyngeal insufficiency, torticollis, nasopharyngeal stenosis, atlantoaxial subluxation (Grisel's syndrome), mandibular condyle fracture, and Eustachian tube injury [4].

In recent years, obstructive breathing has replaced infection as the most common indication for adenotonsillectomy among pediatric patients. With strict adherence to current academic recommendations, a significant portion of patients undergoing adenotonsillectomy because of an obstructive disease may be excluded from outpatient procedural consideration [1].

Changes in surgical techniques over the past years have included widespread transition from adenoid curette use to Punch and Magill forceps and electrocautery Bovie adenoidectomy. Newer techniques are well documented in older patients, and several articles have confirmed that these newer techniques were associated with better outcomes, such as decreased incidences of primary hemorrhage and shorter recovery time. More recent studies that reviewed complications in young patients, considering institutional and personal experiences in operative settings, have caused some surgeons to question the need for overnight admission in young patients [1]. Our study sought to address this need for additional data by retrospectively examining the outcomes of adenoidectomies and adenotonsillectomies performed in 825 children. Specifically, we analyzed postoperative bleeding and minor complications in children undergoing elective adenoidectomy and adenotonsillectomy.

## 2 Materials and Methods

We evaluated the charts of patients who underwent adenotonsillectomy or adenoidectomy from January 2003 to December 2016. All procedures were conducted at our institution after obtaining appropriate institutional review board approval. The study included 825 pediatric patients, aged 1–12 years, with symptoms suggestive of adenoid hypertrophy.

The work has been reported in line with the STROCSS criteria [9].

### 2.1 Data collection

Inclusion criteria were 1–12 years of age the mean age is 1.59, exhibiting one or more of the following symptoms: nasal obstruction, nasal discharge, postnasal discharge, voice change, mouth breathing, earache, decreased hearing, otorrhea, delayed and defective speech, sleep apnea, snoring, hyponasal speech, halitosis, recurrent tonsillitis, otitis media with effusion, upper respiratory tract infection (URTI) it is an infection related to upper resipratory tranct (nose, paranasal sinuses, pharynx, larynx and trachea), asthma, and antibiotic use. We excluded patients under one year old and those above 12 years old. Additionally, we excluded children with past histories of cleft palate repair, bleeding or coagulation defects, and/or craniofacial anomalies.

Patients included in this review were examined for the presence/absence of adenoid facies (an elongated dull expressionless face, prominent mouth, crowded upper teeth, hitched up upper lip, high arched palate, and pinched nose), nasal examination by anterior rhinoscopy, aural examination, oral cavity examination, tympanometry, and X-ray imaging of the nasopharynx (lateral view).

Preoperative nasal endoscopies were performed on all patients. The grading of adenoid hypertrophy based on x-raywas as follows: adenoid tissue filling 1:3 of the vertical height of the choana (mild), adenoid tissue filling 2:3 of nearly (not completely) all choana (moderate), and complete choanal obstruction (severe).

All patients underwent electrocautery Bovie adenoidectomy using the same technique. All surgeries were performed by a single surgeon to avoid intersurgeon variability.

### 2.2 Surgical technique and postoperative care

The patients were intubated under general anesthesia. The postnasal space was examined using a mirror, and adenoidectomy was performed using the electrocautery Bovie technique. Each tonsillectomy was performed using the monopolar technique of 40 W. Both procedures concluded with the reestablishment of proper hemostasis.

For the postoperative care, patients stayed in the recovery room for several hours and were discharged home on the same day. Patients were followed up at the medical center one week after the operation. Postoperative complications such as bleeding " intra-operatively", oropharyngeal malodor, snoring, and fever were monitored throughout the week and graded 1 (mild) 1–3 days, 2 (moderate) 4–6 days, and 3 (severe) 7–10 days. Fever in all patients did not exceed 38.5° C.

### 2.3 Study design and participants

Data were collected from 825 patients, including 344 that received adenotonsillectomies and 480 that received adenoidectomies.

### 2.4 Data analysis

Data were analyzed using the Statistical Package for the Social Sciences (SPSS) [5]. Frequencies and percentages were generated for categorical variables. Associations between categorical variables were tested using either Pearson's, chi-square, or Fisher's exact test. In order to model and assess the association between bleeding complications, noted as a binary outcome (yes/no), and several risk factors, multivariable logistic regression modeling techniques were used to estimate odds ratios (ORs) and their corresponding 95% confidence intervals (95% CIs). All tests performed were two-tailed and probability values (*P*) less than 5% were considered statistically significant.

## 3 Results

### 3.1 Demographics

The prevalence of bleeding complications among patients with adenotonsillectomy was 4.1% (14/344), of whom 11 (78.6%) were males and 3 (21.4%) were females, 7 (50%)were under 3 years old, 4 (28%) were between 4 and 6 years old, and 3 (21%) were above 6 years old. The prevalence of bleeding complications among patients who underwent adenoidectomies were 1.3% (6/480), of whom 3 were females and 3 were males, 5 were under 3 years old, and 1 was above 6 years old. The prevalence of bleeding complications among all patients were 2.43% (20/824), which we determined as remarkably low.

Table 1 presents frequencies of patients according to some risk factors. Male patients were 210 (61%), under six years old (86.1%), taking antibiotics (57.6%), suffering from apnea (75.9%), having no asthma (84.4%), mouth breathers (90.1%), with nasal obstruction (73%), having recurrent tonsillitis (84.6%), with tonsil grade 3 (49.4%), and with severe adenoids on X-ray examination (95.9%).

**Table 1:** Demographic variables for patients who underwent adenotonsillectomy (*n* = 344).

Variable	*n* (%)	Variable	*n* (%)
**Gender**		**Mouth breather**	
Male	210 (61.0)	Yes	310 (90.1)
Female	134 (39.0)	No	34 (9.9)
**Age (years)**		**Nasal obstruction**	
0 to less than 4	155 (45.1)	Yes	251 (73.0)
4 to less than 6	141 (41.0)	No	93 (27.0)
More than 6	48 (14.0)		
**Antibiotic**		**Halitosis**	
Yes	198 (57.6)	Yes	140 (40.7)
No	146 (42.4)	No	204 (59.3)
**Apnea**		**Dysphagia**	
Yes	261 (75.9)	Yes	157 (45.6)
No	83 (24.1)	No	187 (54.4)
**Asthma**		**Recurrent tonsillitis**	
Yes	57 (16.6)	Yes	291 (84.6)
No	287 (83.4)	No	53 (15.4)
**Coughv**		**Ear discharge**	
Yes	56 (16.3)	Yes	62 (18.0)
No	288 (83.7)	No	282 (82.0)
**Runny nose**		**Bleeding complications**	
Yes	141 (41.0)	Yes	14 (4.1)
No	203 (59.0)	No	330 (95.9)
**Tonsil grade**		**Left ear otoscopy**	
1	29 (8.4)	Normal	119 (34.6)
2	77 (22.4)	Wax	70 (20.3)
3	170 (49.4)	Dull	149 (43.3)
4	68 (19.8)	Congested	6 (1.7)
**Right tympanogram**		**URTI**	
A	107 (31.1)	Yes	5 (1.5)
B	215 (62.5)	No	339 (98.5)
C	22 (6.4)		
**Pressure equalization tube**		**Adenotonsillectomy**	
Not done	258 (75.0)	Normal	286 (83.1)
Right	1 (0.3)	Bloody	12 (3.5)
Left	3 (0.9)	Large	18 (5.2)
Bilateral	82 (23.8)	Choanal	24 (7.0)
		Bloody and large	4 (1.2)
**Adenoid X-ray**		**Itchiness**	
Moderate	14 (4.1)	Yes	89 (25.9)
Severe	330 (95.9)	No	255 (74.1)

### 3.2 Major complications

We tested the associations of several covariates with bleeding complications using Pearson's chi-square or Fisher's exact test (Table 2). Ear examination and tonsil grade were significantly associated with bleeding complications. Cough, ear block, and tympanogram approached, but did not achieve, statistical significance (see Table 3).

**Table 2:** Associations between bleeding complications and potential risk factors.

Bleeding complications			
Variable, *n* (%)	Yes	No	*P* value
**Gender**			0.170^a^
Male	11 (5.2)	199 (94.8)	
Female	3 (2.2)	131 (97.8)	
**Age (years)**			0.545^a^
0 to less than 4	7 (4.5)	148 (95.5)	
4 to less than 6	4 (2.8)	137 (97.2)	
More than 6	3 (6.3)	45 (93.8)	
**Antibiotics**			0.559^a^
Yes	7 (3.5)	191 (96.5)	
No	7 (4.8)	139 (95.2)	
**Apnea**			1.00^b^
Yes	11 (4.2)	250 (95.8)	
No	3 (3.6)	80 (96.4)	
**Asthma**			1.00^b^
Yes	2 (3.5)	55 (96.5)	
No	12 (4.2)	275 (95.8)	
**Cough**			**0.059**^b^
Yes	5 (8.9)	51 (91.1)	
No	9 (3.1)	279 (96.9)	
**Runny nose**			**0.210**^a^
Yes	8 (5.7)	133 (94.3)	
No	6 (3.0)	197 (97.0)	
**Nasal obstruction**			0.766^b^
Yes	11 (4.4)	240 (95.6)	
No	3 (3.2)	90 (96.8)	
**Halitosis**			0.867^a^
Yes	6 (4.3)	134 (95.7)	
No	8 (3.9)	196 (96.1)	
**Dysphagia**			0.447^a^
Yes	5 (3.2)	152 (96.8)	
No	9 (4.8)	178 (95.2)	
**URTI**			**0.189**^b^
Yes	1 (20.0)	4 (80.0)	
No	13 (3.8)	326 (96.2)	
**Ear discharge**			1.00^b^
Yes	2 (3.2)	60 (96.8)	
No	12 (4.3)	270 (95.7)	
**Decreased hearing**			0.737^b^
Yes	3 (4.5)	63 (95.5)	
No	11 (4.0)	267 (96.0)	
**Delayed speech**			0.493^b^
Yes	1 (6.3)	15 (93.8)	
No	13 (4.0)	315 (96.0)	
**Ear block**			**0.077**^b^
Yes	5 (8.3)	55 (91.7)	
No	9 (3.2)	275 (96.8)	
**Itchiness**			0.366^b^
Yes	5 (5.6)	84 (94.4)	
No	9 (3.5)	246 (96.5)	
**Left ear otoscopy**			**0.020**^b^
Normal	1 (0.8)	118 (99.2)	
Wax	6 (8.6)	64 (91.4)	
Dull	7 (4.7)	142 (95.3)	
**Otalgia**			0.535^b^
Yes	2 (2.5)	78 (97.5)	
No	12 (4.5)	252 (95.5)	
**Recurrent tonsillitis**			0.460^b^
Yes	11 (3.8)	280 (96.2)	
No	3 (5.7)	50 (94.3)	
**Right tympanogram**			**0.054**^b^
A	1 (0.9)	106 (99.1)	
B	11 (5.1)	204 (94.9)	
C	2 (9.1)	20 (90.9)	
**Recurrent otitis media with effusion**			0.870^a^
Yes	5 (3.8)	125 (96.2)	
No	9 (4.2)	205 (95.8)	
**Tonsil grade**			**0.004**^b^
2	6 (7.8)	71 (92.2)	
3	2 (1.2)	168 (98.8)	
4	6 (8.8)	62 (91.2)	

a Pearson's chi-square test.

b Fisher's exact test.

**Table 3:** Logistic regression modeling of bleeding complications with several potential risk factors, along with their crude and adjusted ORs and 95% CIs.

Variable	Crude OR (CI)	*P* value	Adjusted OR (CI)	*P* value
**Gender**				
Male	1	0.182	1	0.193
Female	0.414 (0.113, 1.513)		0.366 (0.08, 1.666)	
**Age (years)**				
0 to less than 4	1	0.556	1	0.467
4 to less than 6	0.617 (0.177, 2.155)	0.450	0.544 (0.122, 2.417)	0.423
More than 6	1.41 (0.35, 5.675)	0.629	1.505 (0.26, 8.708)	0.648
**Cough**				
No	1	**0.055**	1	0.181
Yes	**3.039** (0.979, 9.439)		2.617 (0.64, 10.70)	
**URTI**				
No	1	0.111	1	0.365
Yes	6.269 (0.654, 60.1)		5.184 (0.147, 182.63)	
**Ear block**				
No	1	**0.077**	1	0.900
Yes	**2.778** (0.897, 8.607)		0.904 (0.188, 4.349)	
**Runny nose**				
No	1		1	0.471
Yes	1.975 (0.67, 5.822)		1.592 (0.45, 5.623)	
**Itchiness**				
No	1	0.395	1	0.872
Yes	1.627 (0.53, 4.99)		1.11 (0.311, 3.965)	
**Left ear otoscopy**				
Normal	1	**0.078**	1	0.315
Wax	11.062 (1.303, 93.91)	**0.028**	4.369 (0.258, 73.98)	0.307
Dull	5.817 (0.706, 47.954)	**0.102**	1.886 (0.101, 35.08)	0.670
**Right tympanogram**				
A	1	0.159	1	0.689
B	5.716 (0.728, 44.869)	0.097	3.308 (0.185, 59.16)	0.416
C	**10.6** (0.917, 122.54)	**0.059**	4.431 (0.128, 154.02)	0.411
**Tonsil grade**				
**2**	1	**0.031**	1	**0.031**
**3**	0.141 (0.028, 0.715)	**0.018**	0.144 (0.027, 0.774)	**0.024**
**4**	1.145 (0.351, 3.733)	**0.822**	1.32 (0.364, 4.782)	0.672

In order to quantify the risk of each covariate, univariate logistic regression was implemented to produce crude ORs and their 95% CIs. Here, patients with tonsil grade 3 were at a reduced risk (86% reduced risk) of developing bleeding complications compared to those with tonsil grade 2 [OR = 0.141, 95% CI: (0.028, 0.715), *P* = 0.018]. Patients with grade C tympanograms had ten times the odds of bleeding complications compared to those with grade A tympanograms [OR = 10.6, 95% CI: (0.917, 122.54), *PV* = 0.054]. Patients with cough had three times the odds of bleeding complications compared to those without cough [OR = 3.03, 95% CI: (0.979, 9.439), *P* = 0.055].

In order to account for any confounding effects, we used a forward selection procedure with logistic regression modeling. All covariates listed in Table 2 were entered in the initial model, and then forward selection was implemented. The *P* values to enter and exit the model were set at 0.15 and 0.20 in order not to exclude any important covariates. The final model is presented in Table 4. Significance was indicated by the omnibus test of model coefficients (chi-square statistic = 26.99, DoF = 10, *P* = 0.003); well-fit data as indicated by Hosmer and Lemeshow test (chi-square = 4.499, DoF = 8, *P* = 0.81), and produced an overall proportion of correct classification of 95.5%.

**Table 4:** Results of logistic regression modeling of bleeding complications with all covariates in Table 2 using a forward selection procedure along with ORs and 95% CIs (*n* = 344).

Variable^a^	β	SE	*P*	OR (95% CI)
**Gender**				
Male	1			
Female	−0.696	0.745	0.35	0.499 (0.116, 2.147)
**Age (years)**				
0 to less than 4	1			
4 to less than 6	−0.879	0.700	0.209	0.415 (0.105, 1.638)
More than 6	−0.095	0.864	0.912	0.909 (0.167, 4.942)
**URTI**				
No	1			
Yes	2.956	1.601	**0.065**	**19.22** (0.834, 443.03)
**Delayed speech**				
No	1			
Yes	1.735	1.288	0.178	5.67 (0.454, 70.74)
**Otalgia**				
No	1			
Yes	−1.605	0.967	**0.097**	**0.20** (0.03, 1.336)
**Left ear otoscopy**				
Normal	1		**0.050**	
Wax	2.805	1.161	**0.016**	**16.53 (1.699, 160.79)**
Dull	2.060	1.136	**0.070**	7.85 (0.847, 72.75)
**Tonsil grade**				
**2**	1		**0.016**	
**3**	−2.172	**0.857**	**0.011**	**0.114 (0.021, 0.611)**
**4**	0.187	**0.654**	0.775	1.206 (0.335, 4.343)

a Results of the forward selection process were adjusted for age and gender.

As indicated in Table 4, otalgia and tonsil grade 3 exerted protective effects against bleeding complications. Our results also indicated that those with URTIs were at an increased risk of bleeding complications [OR = 19.2, 95% CI: (0.834, 443.03)], although this relationship approached, but did not reach, the level of statistical significance (*P* = 0.065). Patients with otalgia were at a reduced risk of having bleeding complications compared to those without otalgia [OR = 0.20, 95% CI: (0.03, 1.336)], although this relationship also approached, but did not achieve, statistical significance (*P* = 0.097). Compared to patients with normal ear examinations, those with wax or dull ears on otoscopy were at a higher risk of bleeding complications [OR = 16.5, 95% CI: (1.69, 160.79) and OR = 7.85, 95% CI: (0.847, 72.75), respectively]. Finally, compared to patients with tonsil grade 1, those with tonsil grade 3 exhibited an 89% reduced risk of bleeding complications [OR = 0.114, 95% CI: (0.021, 0.611), *P* = 0.011].

### 3.3 Minor complications

Examining postadenoidectomy and adenotonsillectomy snoring, 588/825 (71.27%) patients had no snoring, 208 patients (25.21%) had grade 1, 18 patients had grade 2 (2.18%), and 11 patients (1.33%) had grade 3. No malodor was noted in 586/825 patients (71.03%); however, 192 patients (23.27%) had grade 1, 37 patients (4.48%) had grade 2, and no patients experienced grade 3 malodor. Lastly, 660/825 patients (80%) had no postoperative fever, 107 patients (12.97%) had grade 1 fever, 57 patients (6.90%) had grade 2 fever, and no patients exhibited grade 3 fever.

## 4 Discussion

Adenoidectomy is considered to be a safe and minor procedure and is usually done alone or accompanied by tonsillectomy. There are many methods used for adenoidectomy but, for the purpose of our study, we examined patients who underwent the electrocautery Bovie technique. The aim of our study was to determine the prevalence of postadenoidectomy or adenotonsillectomy bleeding and minor complications including snoring, malodor, and fever, as well as associated factors that are correlated to such complications.

In our study, postoperative primary hemorrhage in adenoidectomy occurred in 2.43% (20/824) of the patients, which is remarkably low. As for adenotonsillectomy, primary hemorrhage was observed in 4.1% (14/344). These results were similar to those of Richmond et al. [6] and Valtonen et al. [7] in that adenotonsillectomy was associated with a higher prevalence of bleeding than adenoidectomy alone. Other risk factors that appeared to increase the risk of bleeding (intraoperative primary hemorrhage) were the history of abnormal type C tympanogram, patients with lower-grade (grade 2) tonsils, and those with URTIs.

Lesinskas et al. [8] noted that adenoidal regrowth occurred more often in children who were treated postoperatively with antibiotics, and in postoperative settings, an association was noted between increased antibiotic consumption and adenoidal regrowth. These results stand in contrast to those found in our study, where 57% of the patients were treated with multiple antibiotics preoperatively and no patients experienced regrowth or needed a revision surgery.

Notably, we reviewed the incidence of minor postoperative complications including malodor, snoring, and fever that did not exceed 38.5 °C. These data have not been reported previously.

## 5 Conclusions

In conclusion, although many studies and articles have examined the prevalence of major complications such as bleeding, none have reviewed minor complications that patients usually experience during the first few days postoperatively. We found that almost 4% of the patients developed bleeding after adenotonsillectomy and only 2% of the patients had bleeding alone after adenoidectomy. On the other hand, 15–25% of the patients developed minor complications including snoring, malodor, and fever, independent of their preoperative symptoms.

## Compliance with ethical standards

### Funding

No sources of funding were received for this research.

### Ethical approval

All procedures performed in studies involving human participants were in accordance with the ethical standards of the institution.

Ethical approval has been exempted by out institution.

### Authors’ contributions

Dr Khalid; data analysis and contribution.

Dr Jumana; data collection and analysis, writing the paper.

Dr Ahmed; data analysis and contribution.

### Conflicts of interest

None of the authors has any conflict of interest to declare.

### Trial registry number

researchregistry4129.

### Guarantor

Dr. Jumana Hussain.

### Provenance and peer review

Not commissioned, peer reviewed.

## Appendix A Supplementary data

STORCSS STORCSS
